# Voluntary Imitation in Alzheimer’s Disease Patients

**DOI:** 10.3389/fnagi.2016.00048

**Published:** 2016-03-07

**Authors:** Ambra Bisio, Matthieu Casteran, Yves Ballay, Patrick Manckoundia, France Mourey, Thierry Pozzo

**Affiliations:** ^1^Department of Robotics, Brain and Cognitive Sciences, Istituto Italiano di TecnologiaGenoa, Italy; ^2^Department of Experimental Medicine, University of GenoaGenoa, Italy; ^3^INSERM U1093 Cognition, Action et Plasticité Sensorimotrice, Université de Bourgogne Franche-ComtéDijon, France; ^4^Service de Médecine Interne Gériatrie, Hôpital de Champmaillot, Centre Hospitalier UniversitaireDijon, France; ^5^Institut Universitaire de France, Université de Bourgogne Franche-ComtéDijon, France

**Keywords:** motor imitation, action observation, movement execution, Alzheimer’s disease, social interaction

## Abstract

Although Alzheimer’s disease (AD) primarily manifests as cognitive deficits, the implicit sensorimotor processes that underlie social interactions, such as automatic imitation, seem to be preserved in mild and moderate stages of the disease, as is the ability to communicate with other persons. Nevertheless, when AD patients face more challenging tasks, which do not rely on automatic processes but on explicit voluntary mechanisms and require the patient to pay attention to external events, the cognitive deficits resulting from the disease might negatively affect patients’ behavior. The aim of the present study was to investigate whether voluntary motor imitation, i.e., a volitional mechanism that involves observing another person’s action and translating this perception into one’s own action, was affected in patients with AD. Further, we tested whether this ability was modulated by the nature of the observed stimulus by comparing the ability to reproduce the kinematic features of a human demonstrator with that of a computerized-stimulus. AD patients showed an intact ability to reproduce the velocity of the observed movements, particularly when the stimulus was a human agent. This result suggests that high-level cognitive processes involved in voluntary imitation might be preserved in mild and moderate stages of AD and that voluntary imitation abilities might benefit from the implicit interpersonal communication established between the patient and the human demonstrator.

## Introduction

In mild and moderate stages of Alzheimer’s disease (AD), despite cognitive decline, some basic mechanisms, such as motor resonance—i.e., automatic activation, during actions perception, of the perceiver’s motor system (Rizzolatti et al., 1999), which are believed to underlie natural and spontaneous interaction among humans (Chartrand and Bargh, 1999)—seem to be preserved. In a recent article we showed that the observation of an abstract moving stimulus influenced the motor responses of AD patients (Bisio et al., 2012) suggesting the preservation of motor resonance mechanisms, expressed in the form of automatic imitation, which is the involuntary tendency of humans to copy the features of the observed actions (Heyes, 2011). However, when AD patients face more challenging tasks, which do not rely only on automatic processes but also on explicit voluntary mechanisms and require the patient to pay attention to the external events, the cognitive deficits resulting from the disease might negatively affect patient’s behavior. This might be the case for voluntary imitation, a social-cognitive mechanism (Korman et al., 2015), which involves observing another person’s action and translating explicitly these precepts into one’s own actions (Brass and Heyes, 2005).

Voluntary motor imitation makes it possible to interact with others by volitionally sharing behavioral states. It is a powerful biological resource for cognitive development (Meltzoff and Moore, 1977), social interaction (Chartrand and Bargh, 1999) and motor learning (Byrne and Russon, 1998). Altered imitation processes might result in abnormal behavior, as in the case of compulsive imitation behavior (Lhermitte et al., 1986; Pandey and Sarma, 2015). Another pathological condition related to imitation mechanisms is apraxia, defined as the difficulty to produce gestures on verbal command or by imitation. These are examples of the non-memory cognitive symptoms of AD. Several studies that characterized the relationship between disease progression and the different kinds of apraxia generated contrasting results (Edwards et al., 1991; Travniczek-Marterer et al., 1993). While some researchers reported more difficulties to execute transitive gestures (Rapcsak et al., 1989), others showed that AD patients were mostly impaired in the imitation of pantomimes and meaningless (intransitive) movements, the latter explained as being caused by impaired visual-spatial analysis (Rousseaux et al., 2012).

The ability to voluntarily imitate the actions of others could thus be negatively affected by visual deficiencies since it requires intense visuospatial processing and the ability to match the model’s posture and movements with one’s own motor repertoire (Goldenberg, 1999). Furthermore, it is known that AD patients exhibit attention deficits, which particularly affect their ability to focus on stimulus modifications and to follow elementary instructions (Perry and Hodges, 1999), as well as that to imitate a specific feature of the observed motion. Another source of difficulty in voluntary imitation could be the kind of stimulus AD patients have to reproduce. Indeed, AD is also a source of visual dysfunction, such as color discrimination (Cronin-Golomb et al., 1993) and shape recognition, especially when objects or persons are moving (Rizzo and Nawrot, 1998). Consequently, one can expect the imitation of movement features to be impaired in the presence of an enriched stimulus, as in the case of a real person moving in front of the patient, as compared with a simple digitalized visual display. Nevertheless, when AD patients watch actions performed by a human being, socioemotional processes (Narme et al., 2013) might intervene to improve their ability to relate to and maybe to voluntarily imitate the observed movement’s kinematic features. Indeed, it has been shown that the ability to identify social and emotional signals and to access social knowledge is intact in patients with mild and moderate stages of AD, but impaired in other forms of dementia (Shany-Ur and Rankin, 2011).

In the present study we asked AD patients and healthy age-matched participants to voluntarily reproduce the kinematic features of two moving stimuli. Precisely, subjects were required to observe and to reproduce the velocity of an abstract, computer-generated stimulus (a dot moving upwards on a screen) and of a human demonstrator, who was facing the participants and performed upward pointing movements with his right arm. In view of the aforementioned considerations, one could expect either the loss or the preservation of AD patients’ capacity to reproduce the velocity of the observed stimuli. In the latter case, the preserved motor resonance could have enabled the voluntary imitation. Furthermore, comparing the imitation performance of AD patients when they looked at a simple dot with that when they looked at a human demonstrator would shed light on their ability to select and reproduce a precise kinematic schema of the observed motion, even in the case of an enriched stimulus as a human being.

## Materials and Methods

### Participants

The experimental group was composed of 23 elderly participants (14 women), from 75 to 86 years of age (mean age, 82 ± 5), with mild or moderate AD diagnosed according to the French National Institute of Neurology and Communication Disorders and Strokes—The Alzheimer’s Disease and related Disorders Association (NINCDS-ADRDA) and the Diagnostic and Statistical manual-IV-Text Revised (DSM IV-TR) criteria, and living at home or in a nursing home specializing in AD. They did not differ according to their residence. They all underwent a comprehensive diagnostic evaluation, which included a clinical assessment, detailed neuropsychology tests, brain magnetic resonance imaging (MRI) and an examination of motor competencies. All of them presented progressive cognitive impairment, predominantly affecting memory and no evident problem in motor performances. Their Mini-Mental State Examination (MMSE) scores were between 12 and 23 (mean, 17 ± 4). The control group (CG) was composed of 14 healthy participants (10 women and 4 men), from 75 to 87 years of age (mean age, 82 ± 4), living at home. Their MMSE scores were between 25 and 30 (mean, 29.5 ± 1.5). All participants were right-handed, and had normal or corrected-to-normal vision. They were able to hear adequately, to pay attention to the examiner’s behavior and to understand elementary questions. Written informed consent was obtained from each participant or their guardians, and the Local Ethics Committee of Burgundy Hospitals (Dijon University Hospital—CHU-CMRR-France) approved the protocol. Table 1 contains the demographic data.

**Table 1:** **Demographic data of the Alzheimer’s disease (AD) patients and healthy age-matched participants (control group, CG)**.

	AD	CG	Statistics
Number of subjects	23 (14 females)	14 (10 females)	not significant
Age (mean years ± SD)	82 ± 5	82 ± 4	not significant
Range	75 ÷ 86	75 ÷ 87	
MMSE (mean ± SD)	17 ± 4	29.5 ± 1.5	*p* ≪ 0.01
Range	12 ÷ 23	25 ÷ 30	
Education (mean years ± SD)	5.07 ± 1.82	5.21 ± 1.63	not significant
Range	3 ÷ 8	3 ÷ 8	

### Materials and Procedure

**Figure 1:**
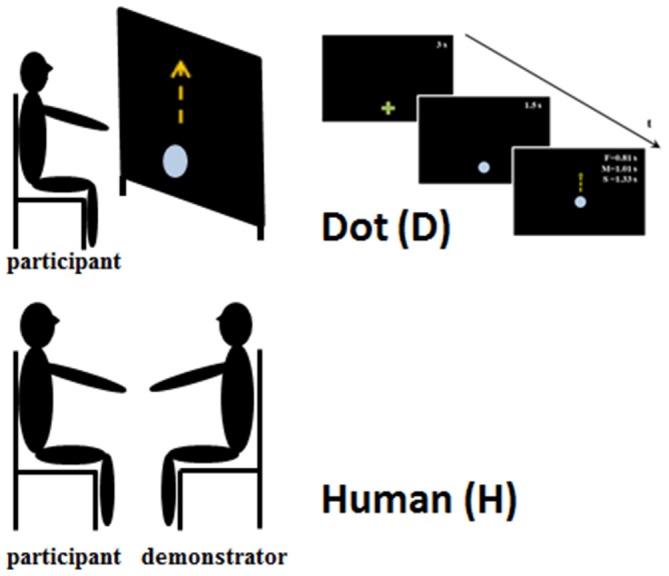
**Experimental set-up.** The upper part of the figure represents the set-up when the participants observed the Dot stimulus. They were seated in front of a large screen and they observed the upward displacement of the Dot at three different speeds and durations: slow—S 1.33 s; medium—M 1.01 s; and fast—F 0.81 s. The bottom part of the figure shows the experimental set-up for the human obsevation condition, in which the participants and human demonstrator were seated face to face. In both conditions participants were requested to observe the stimulus movements and when the stimulus stoped to reach its/his final position, imitating its/his velocity.

The experiment lasted about 20 min. A moving stimulus was used as a template to test the effect of motion perception on the execution of subsequent pointing movements. Participants received verbal feedback during the testing procedure in order to remind them about the experimental instructions. The beginning of each phase was preceded by a training step, which ended when the participant understood the task. The experimental set up is represented in Figure 1.

### Dot Observation (D)

The experiment was performed in a darkened room. Participants sat on a chair, in front of a large rear projection screen (170 × 230 cm) at a viewing distance of about 60 cm. A video-projector, with a refresh rate of 60 Hz, placed behind the screen and connected to a PC, back-projected the visual stimuli onto the display screen. The visual stimulus was generated using MatLab Psychtoolbox 3 (Brainard, 1997). An optoelectronic system (SMART) with five cameras was used to record movements at a sampling frequency of 120 Hz. One passive infrared reflective marker was applied onto the tip of the participant’s right index finger.

A green cross was displayed at the movement starting position. After 3 s, the green cross was replaced by a light blue dot (3.2 cm in diameter). The dot was displayed in this position for 1.5 s, and then started to move vertically, over a distance of 52 cm. Dot motion reproduced the kinematic characteristics of a human vertical movement. The dot moved at three different mean velocities: slow (0.39 m/s, corresponding to 1.33 s), medium (0.51 m/s, corresponding to a total time of 1.01 s), and fast (0.64 m/s, corresponding to 0.81 s). Stimulus velocities were randomized. Participants were asked to point at the green cross, then to watch the movement of the dot until it reached its final (visible) position. When the dot stopped, the participants replicated its movement. We asked the participants to wait until the stimulus stopped to start moving because in such a way they could better appreciate the kinematic feature of the observed movement. Particular emphasis was given to the imitation of the dot velocity. The test was repeated four times for each dot velocity (12 trials in total; Figure 1, upper part).

### Human Observation (H)

The person that acted as the stimulus (human demonstrator, H) was a young man and was the same in all the experiments. He was previously trained to make vertical straight movements at three different velocities (V_H_, Slow, Medium and Fast) with his right arm kept in a comfortable position. As in the case of the dot, the human demonstrator’s velocities were randomized. The demonstrator and the participant sat on comfortable chairs facing each other. The participants were instructed to point their right index finger at the demonstrator’s fingertip, then to watch the movement until it stopped. They then replicated the movement, to reach the demonstrator’s final fingertip position. Also in this condition particular emphasis was given to the imitation of the demonstrator’s movement velocity. Each participant accomplished 12 movements.

### Data Treatment

#### Data Processing

Kinematic data were low-pass filtered at 5 Hz using a 2nd order Butterworth filter. To define the onset and offset of the movement, we chose a threshold corresponding to 5% of the maximum value of the movement velocity profile.

#### Data Analysis

In order to provide a quantitative description of both the planning and execution phases of participants’ movement, reaction time (RT) and mean velocity were considered the outcome variables for all trials.

RT was computed as the difference in time between the end of the stimulus motion and the start of the participant’s pointing movement. In order to check participants’ ability to suppress the motor response until the stimulus stopped, the percentage of RT > 0 for each participant after the observation of both D and H was calculated. These values were statistically evaluated using a mixed-design analysis of variance (ANOVA) with Group (2 levels, CG and AD), as the between-subject factor, and Stimulus (2 levels, D and H), as the within-subject factor.

Participants’ V_P_ values were compared using a mixed-model analysis of variance with Group (2 levels, CG and AD) as the between-subject factor, and velocity as the within-subject factor (3 levels, Slow, Medium and Fast). This statistical analysis method was chosen for its flexibility to designs that are not perfectly balanced, as in our case. Moreover, it allowed us to take into account the intrinsic (and uncontrolled) variability among the participants, which was considered everywhere as a random factor. Because of the differences between the dot (D) and the demonstrator’s (H) velocities, two distinctive mixed-model analyses of variance were performed on D and H datasets. In the H condition, the data were classified on the basis of demonstrator’s movement velocity (V_H_): i.e., Slow V_H_ < 0.4, Medium 0.4 < V_H_ < 0.7 and Fast V_H_ > 0.7.

A linear regression model illustrated the relationship between V_D,H_ and V_P_ for each participant. The slope of the linear fits was mainly used to evaluate whether and how much participants were able to replicate the stimulus’ velocity and can be considered a measure of the accuracy of the imitation. Slope = 1 indicated the perfect reproduction of the stimulus velocity. A mixed-designed ANOVA, with Group as the between-subjects factor, and Stimulus (2 levels, D and H), as the with-in subjects factor, was applied on slope values. Significant interactions were always interpreted with Newman-Keuls *post hoc* comparisons.

Finally, we computed another parameter, which provided information related to participants’ imitation ability. This parameter was obtained for each subject by subtracting the slope value of the linear regression model applied to describe the relationship between participant’s and stimuli velocities from the slope of the perfect imitation line and calculating the absolute value of this difference (*abs(1−slope)*); the greater the difference the poorer the imitation performance. Pearson’s correlation was applied to assess any correlation between the imitation abilities defined by this index and participants’ cognitive status, assessed by the MMSE score.

## Results

All participants completed the experiment. According to an informal interview made at the end of the experiment, none had problems seeing the visual stimuli and none considered the task difficult.

**Figure 2:**
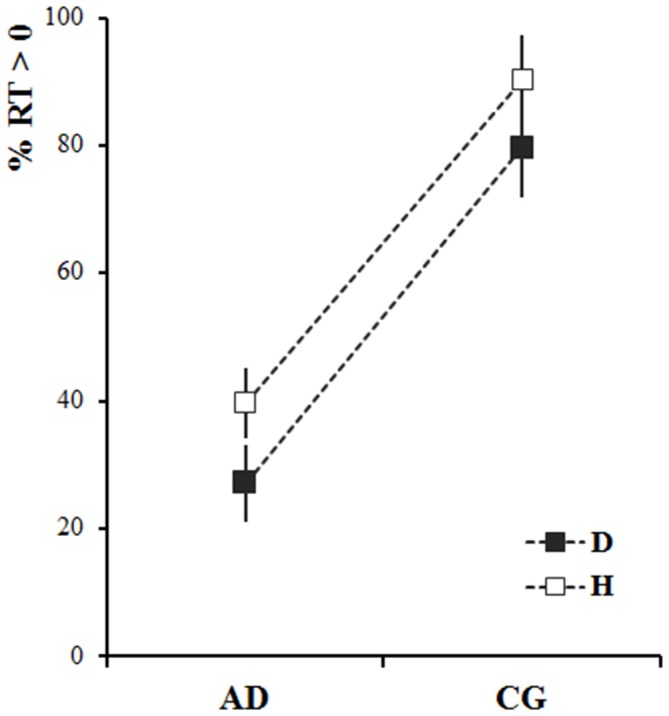
**Percentage of non-anticipated responses (reaction time, RT > 0) for Alzheimer’s disease patients (AD) and healthy elderly participants (control group, CG), after observing the dot (D-black) and the human demonstrator (H-white).** The squares represent the average values calculated across the participants and stimuli velocities. The vertical bars represent the standard errors.

Figure 2 shows the mean percentage of participants’ responses that started after the end of stimulus motion (i.e., RT > 0) as requested by the experimenter. For both dot stimulus and human demonstrator, most healthy participants (CG) were able to wait until the end of the stimulus movement before starting to move (around 80% of positive RT values). In contrast, most of the AD patients’ responses were anticipated (RT < 0): i.e., they started to move while the stimulus was still moving. Moreover, when the stimulus was the human demonstrator both groups were better able to follow the instructions than when the stimulus was the dot. These observations were confirmed by the statistical analysis. The results of the mixed-design ANOVA showed a significant Group effect (the percentage of anticipated responses in AD patients was significantly higher than that in the CG—*F*_(1,36)_ = 47.19, *p* < 0.001) and Stimulus effect (the percentage of anticipated responses was lower in both groups when they observed the human demonstrator—*F*_(1,36)_ = 6.31, *p* < 0.05).

**Figure 3:**
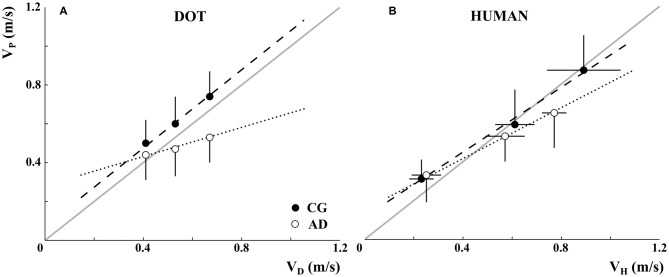
**Alzheimer’s disease patients’ (AD-white) and healthy elderly participants’ (control group, CG-black) mean velocities (V_P_, *y-axis*) as a function of dot velocity (V_D_, *A; x-axis*) and human demonstrator’s velocity (V_H_, *B; x-axis*).** The data refer to the mean velocities over movement repetitions and the vertical error bar to the standard deviation. It can be noted that the demonstrator’s velocities were actually inaccurate (see the horizontal error bars representing standard deviations). Dotted and dashed lines represent the linear relationship between AD and CG participants and V_D,H_, while the gray lines correspond to a perfect imitation of the stimuli velocity.

The mean pointing velocities of participants in the CG and AD patients (V_P_, black and white dots, respectively) are represented in Figure 3 as a function of the stimuli velocities (V_D_, Figure 3A and V_H_, Figure 3B). The participants of both groups understood the experimental instructions concerning the imitation of the stimuli velocities. Participants’ velocities changed in accordance with the dot and demonstrator’s velocities. However, this effect was less pronounced for AD patients when they observed the dot. Indeed, AD patients’ movement velocities were closer to stimulus velocities when the stimulus was a human demonstrator than when it was a dot.

A significant Group*Velocity interaction emerged in data associated with observation of the dot (*F*_(2,71.72)_ = 17.05, *p* < 0.001). The subsequent *post hoc* comparison showed that AD patients’ mean velocities for Fast and Slow stimuli differed significantly (*p* < 0.001). Further, the mean velocities of the CG at each stimulus velocity were significantly different one from the other (*p* < 0.001). In addition, the movements performed by the participants in the CG in the Medium and Fast conditions were significantly faster than AD patients’ movements (*p* < 0.001).

The statistical analysis of the data recorded during the human demonstrator condition showed a combined effect of Group and Velocity (Group*Velocity: *F*_(2,51.35)_ = 3.88, *p* < 0.05). *Post hoc* comparison showed that for both groups the velocity in Fast condition was higher than in Medium and Slow conditions, and that in the Medium condition was higher than in Slow (always *p* < 0.001). Moreover, a significant difference between patients’ and participants’ mean velocities appeared with regard to Fast and Medium stimuli (*p* < 0.001); this result was probably related to the gap between the human demonstrator’s velocities in Fast conditions observed by the two groups (see Figure 3B).

**Figure 4:**
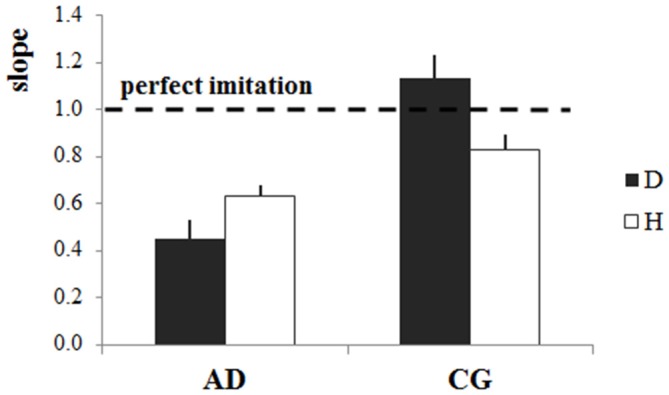
**Accuracy of imitation: slopes of the linear regression model.** Mean slope values (±stardard error) for each stimulus (dot-D and human demonstrator-H) and for both groups (Alzheimer’s disease patients—AD, control group—CG). The horizontal dotted line indicates the perfect imitation of the stimulus velocity, and refers to the slope of the perfect imitation line in Figure 2.

ANOVA comparing the slope values (i.e., index of the accuracy of the imitation) in the four experimental conditions showed a significant Group*Stimulus interaction (*F*_(1,35)_ = 14.36, *p* = 0.0006; Figure 4). Concerning the results of the post doc analysis, slope values for the CG were significantly higher when participants observed the dot than when they observed the human demonstrator (*p* = 0.002). The mean slope value for the dot stimulus slightly exceeded 1 (slope_D_ = 1.13), whereas it was lower than 1 for the human demonstrator (slope_H_ = 0.83). However, if we consider the absolute difference between the perfect imitation and the slope values in the two cases there was only a marginal divergence from 1 (D: 0.13, H:0.17), which cannot be interpreted as a significant difference in the imitation performance. Concerning AD patients, slope values were significantly higher with the human demonstrator than with the dot stimulus (*p* = 0.045). In addition, whereas AD patients’ slope values were significantly lower those of the CG for the dot stimulus (*p* = 0.0001), there were no differences between the slopes values of the two groups for the human demonstrator (*p* = 0.12), meaning that AD patients imitation performance reached that of the CG when there was a human demonstrator.

**Figure 5:**
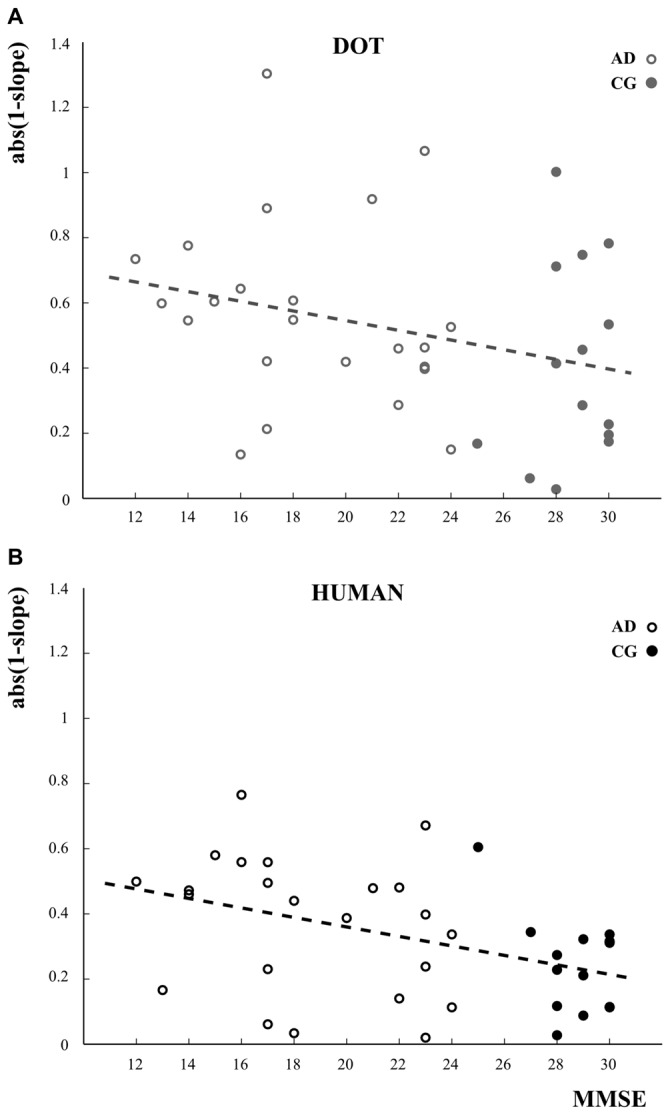
**Linear relationships between the imitation index and the cognitive status assessed using the mini mental state examination (MMSE) scores for the dot condition (A, gray circles) and the human demonstrator condition (B, black circles).** Open and closed circles refer to the data of AD patients and the control participants (CG), respectively. The imitation index is here defined as the absolute difference between the slope of the perfect imitation line (slope = 1) and the slope of the linear regression model applied to describe the relationship between participants’ and stimulus velocities. The greater the difference, the worse the imitation performances.

Correlation analysis between the imitation index defined as *abs(1−slope*) and MMSE showed that the more severe the cognitive impairement (low MMSE) the worse the imitation abilities (high values of *abs(1−slope)*). Whereas this result was only a trend for the dot stimulus (*R* = −0.28, *p* = 0.09; Figure 5A), there was a significant negative correlation between the imitation abilities and MMSE when participants oberved the human demonstrator (*R* = −0.42, *p* = 0.009; Figure 5B).

## Discussion

This study aimed to determine AD patients’ ability to imitate the kinematic features of two kinds of stimuli: an abstract, computer-generated display (a dot) projected on a large screen, and a human demonstrator facing the participant. The main findings were that: (1) AD patients were able to imitate the kinematic features of the stimuli; and (2) the imitation performance was better when they observed the human demonstrator than when the stimulus was abstract. Furthermore, the imitation performance worsened with increasing cognitive impairment. Finally, the percentage of anticipated motor responses with respect to the end of the stimulus movement was lower when the stimulus was a human demonstrator.

### The Nature of the Stimulus Modulated the Start of the Motor Response

The present data showed that most healthy participants were able to follow the instruction given by the experimenter about the starting time (i.e., to wait until the stimulus stopped before starting the movement) whereas most AD patients started to move before the end of stimulus motion. This result confirms our previous work which showed that in a context of implicit automatic imitation, AD patients were not only influenced by the velocity of the observed stimulus, but were compulsively attracted by the display and could not refrain from moving (Bisio et al., 2012). This indicates that the mere presence of a moving stimulus was sufficient to trigger the start of movement in AD patients. As previously suggested (Bisio et al., 2012), the inability to prevent movement when observing a moving stimulus could reflect inadequate functioning of the inhibitory mechanisms. This dysfunction could be due to altered cortico-cortical connections linking both the basal forebrain system and the parietal lobes to the frontal lobes (Lhermitte et al., 1986; Aron et al., 2003).

Intriguingly, in both groups, the percentage of non-anticipated responses (i.e., RT > 0) increased when participants looked at the human demonstrator. This effect might be explained by the hypothesis that, compared to a simple dot moving on a screen, a human demonstrator is a more salient and meaningful stimulus in the eyes of the participants. Therefore, by delaying movement onset so as to catch the details of his motion, they could better fulfil the task.

### The Interaction with a Human Demonstrator Boosted Alzheimer Patients’ Imitation Performance

Both healthy elderly participants and AD patients complied with the experimental instruction concerning the imitation of the stimulus velocity: indeed, the velocity of participants’ pointing movements increased with increasing stimulus velocity (both D and H). Thus, participants were able to imitate both an abstract computer-generated stimulus and a human demonstrator. Since deficits in motor planning (Edwards et al., 1991; Travniczek-Marterer et al., 1993), attention mechanisms (Perry and Hodges, 1999), visuomotor integration (Gilmore et al., 1994; Rizzo and Nawrot, 1998; Tippett and Sergio, 2006; Tippett et al., 2007), and movement control (Ghilardi et al., 1999) have been reported in individuals with AD-type dementia, this finding was not easily predictable. Our result implies that AD patients understood the experimental instructions, extracted the correct information from the stimulus (i.e., position and velocity), used that information to plan their motor response, and executed the movement imitating the stimulus velocity. Therefore, the high-level cognitive processes that underlie all of these functions seem to be preserved in the mild and moderate phases of AD and could be exploited during training programs and cognitive rehabilitative interventions. This result also suggests that the kinematic representation of action (e.g., velocity, duration and spatial trajectory) is preserved during pathological (AD) aging. More speculatively, the preserved ability to match the kinematic features of the visual model with the internal motor repertoire could have driven the initial planning phase of AD motor response, and helped participants to voluntarily replicate the kinematics of the model.

Interestingly, participants’ voluntary imitation abilities varied depending on whether they observed the dot or the human demonstrator. The ability of CG participants to reproduce the observed kinematics was not stimulus dependent. This finding is in agreement with those reported in our previous studies (Bisio et al., 2010, 2012, 2014) and could reflect the participants’ ability to extract low-level features of motion (i.e., kinematic details of the stimulus) without being influenced by other features of the stimulus not related to the movement itself.

AD patients’ behavior contrasted with that of CG participants. Remarkably, although AD individuals varied their movement velocity when velocity of the abstract stimulus changed, the imitation performance improved when they observed a human demonstrator. One hypothesis to explain this effect is that the observation of human movement not only induced an automatic match between the observed movement kinematics and the patient’s internal motor repertoire, but also triggered emotional mechanisms associated with social interaction. Generally, in its initial and moderate stages, AD is dominated by cognitive symptoms, whereas social and emotional functioning is relatively spared (Rosen et al., 2004; Mendez et al., 2005; Rankin et al., 2008) and patients do not exhibit increased levels of interpersonal dysfunction (Rankin et al., 2003). Furthermore, it has been suggested that while AD may impair cognitive processes, the capacity for emotional communication is preserved (Rankin et al., 2008; Shany-Ur and Rankin, 2011). Faces, bodies, and particularly dynamic body stimuli, carry precious information on the actions, intentions and emotional states of others (de Gelder et al., 2014). Therefore, the perception of this kind of information might have helped patients to accomplish the task, thus leading to a better imitation performance compared with that achieved during dot observation. More speculatively, according to the theories of embodied cognition, which link the cognitive processing of an event to its sensory and motor components, the emotional content of the interaction between AD patients and the human demonstrator might have enhanced their imitation abilities (Vallet, 2015).

From a neurophysiological point of view, the automatic imitation of the observed movement features suggests that the activity of a circuit which involves the mirror neuron system, the superior temporal sulcus (Iacoboni et al., 1999) and the sensorimotor areas for movement production is preserved. Preservation of the ability to voluntarily imitate movement, as tested here, entails the functioning of a broader neural network that also includes sub-cortical regions, such as the basal ganglia, for the decision making component. Nevertheless, the occurrence of compensatory mechanisms where the intact brain regions take over the functions of the injured areas cannot be ruled out (Buckner, 2004; Nithianantharajah and Hannan, 2009). Unfortunately, we are not able to provide a complete description of the results of the neuroimaging and neuropsychological investigations the patients underwent. Therefore, these interpretations remain speculative.

### The Cognitive Status Affected Imitation Performance

We found that the cognitive status influenced participants’ imitation performance. Indeed, the existence of a negative correlation between the voluntary imitation abilities and participants’ MMSE shows that increasing cognitive impairment reduces imitation ability. This could reflect a limited comprehension of the experimental instructions in the moderate stage of the pathology. In this case one may have expected a similar result for the two stimuli. However, we found that the relationship became significant only when observing the demonstrator, suggesting more than a difficulty to understand the experimental instruction. Indeed, it is likely that the decrease in the MMSE score was also related to progression of the AD in other fields. Decreasing visual accuracy, less motivation to interact with others, or degradation of the neural networks responsible for imitation are some potential candidates among several others accounting for this result. Future studies will be necessary to investigate this issue in depth.

## Conclusion

The main finding of the present study is that AD patients were able to voluntarily imitate the kinematic features of a moving stimulus, an ability that improved when watching the human demonstrator compared to the computerized object. The present findings may be clinically relevant for cognitive interventions, especially when the efficacy of computer-based techniques is compared with that of traditional training programs, where the therapist plays an active role. These innovative computer-based methodologies have been proposed for cognitive training (Hofmann et al., 1996, 2003), stimulation (Tárraga et al., 2006), and rehabilitation treatments (Cipriani et al., 2006). However, the effectiveness of these techniques, and in particular the improvements they could bring to daily-life activities, is still greatly debated (Bahar-Fuchs et al., 2013). In the present work, by voluntarily imitating the dot velocity, AD patients exhibited their ability to interact with a computerized system, encouraging the use of computerized exercises in cognitive interventions. Nevertheless, the improvement in imitation performance when patients faced the demonstrator suggests that the presence of a human agent could increase the efficacy of the treatment.

## Author Contributions

AB and TP conceived and designed the experiments. AB, MC, YB, and QW performed the experiments. AB, MC, YB and TP analyzed the data. AB, FM, PM and TP interpreted the data. FM, PM and TP contributed the materials. AB and TP wrote the article. MC, YB, FM, PM and TP revised the article. MC, YB, FM, PM and TP gave the final approval.

## Conflict of Interest Statement

The authors declare that the research was conducted in the absence of any commercial or financial relationships that could be construed as a potential conflict of interest.

## Abbreviations

AD: Alzheimer’s disease
CG: Control group
D: Dot
H: Human demonstrator
F: Fast
M: Medium
S: Slow
RT: Reaction Time
V_P_: Participants’ velocity
V_H_: Human demonstrator’s velocity
V_D_: Dot velocity.
